# Sclerosing Polycystic Adenosis of the Parotid Gland: A Case Report and Literature Review

**DOI:** 10.7759/cureus.86004

**Published:** 2025-06-14

**Authors:** Marouane Imdary, Najib Elorfi, Oumaima Lakhal, Othmane Benhoummad, Mehdi Elfakiri

**Affiliations:** 1 Otolaryngology Department, Centre Hospitalo-Universitaire (CHU) Souss Massa, Agadir, MAR; 2 Otolaryngology-Head and Neck Surgery, Centre Hospitalo-Universitaire (CHU) Souss Massa, Agadir, MAR

**Keywords:** benign salivary gland lesions, parotidectomy, salivary gland tumors, sclerosing polycystic adenosis, standard parotidectomy incision

## Abstract

Sclerosing polycystic adenosis (SPA) is a rare benign salivary gland lesion that resembles both benign and malignant tumors, complicating diagnosis. We present the case of a 44-year-old woman with progressive right lateral facial swelling. MRI suggested a pleomorphic adenoma and initial parotidectomy was followed by total excision based on frozen section results. Final histopathological analysis confirmed SPA, with characteristic abnormal glandular architecture in a sclerotic stroma and absence of malignant features. Complete surgical excision was curative. This case underscores the importance of considering SPA in the differential diagnosis of parotid masses.

## Introduction

Sclerosing polycystic adenosis (SPA) is a rare lesion of the salivary glands, first identified in 1996 by Smith et al. [1,2]. Its anatomical and clinical features are similar to those of benign and complex breast lesions, such as cystic mastitis. Although its main location is the parotid gland, cases have also been reported in the submandibular and accessory salivary glands.

Histopathological similarities with certain malignant tumors, particularly carcinomas, complicate diagnosis, making immunohistochemical studies necessary [3]. Treatment is primarily surgical, and the prognosis is generally favorable. We report a case of sclerosing polycystic adenosis of the parotid gland in a 44-year-old woman.

## Case presentation

The patient is a 44-year-old housewife with a history of hypertension since 2021, under treatment. Her symptoms began four years earlier with the progressive appearance of a lateral facial swelling over the right parotid region, associated with pain during mastication and referred otalgia, without other accompanying signs, evolving in an afebrile context with the preserved general condition.

**Figure 1:**
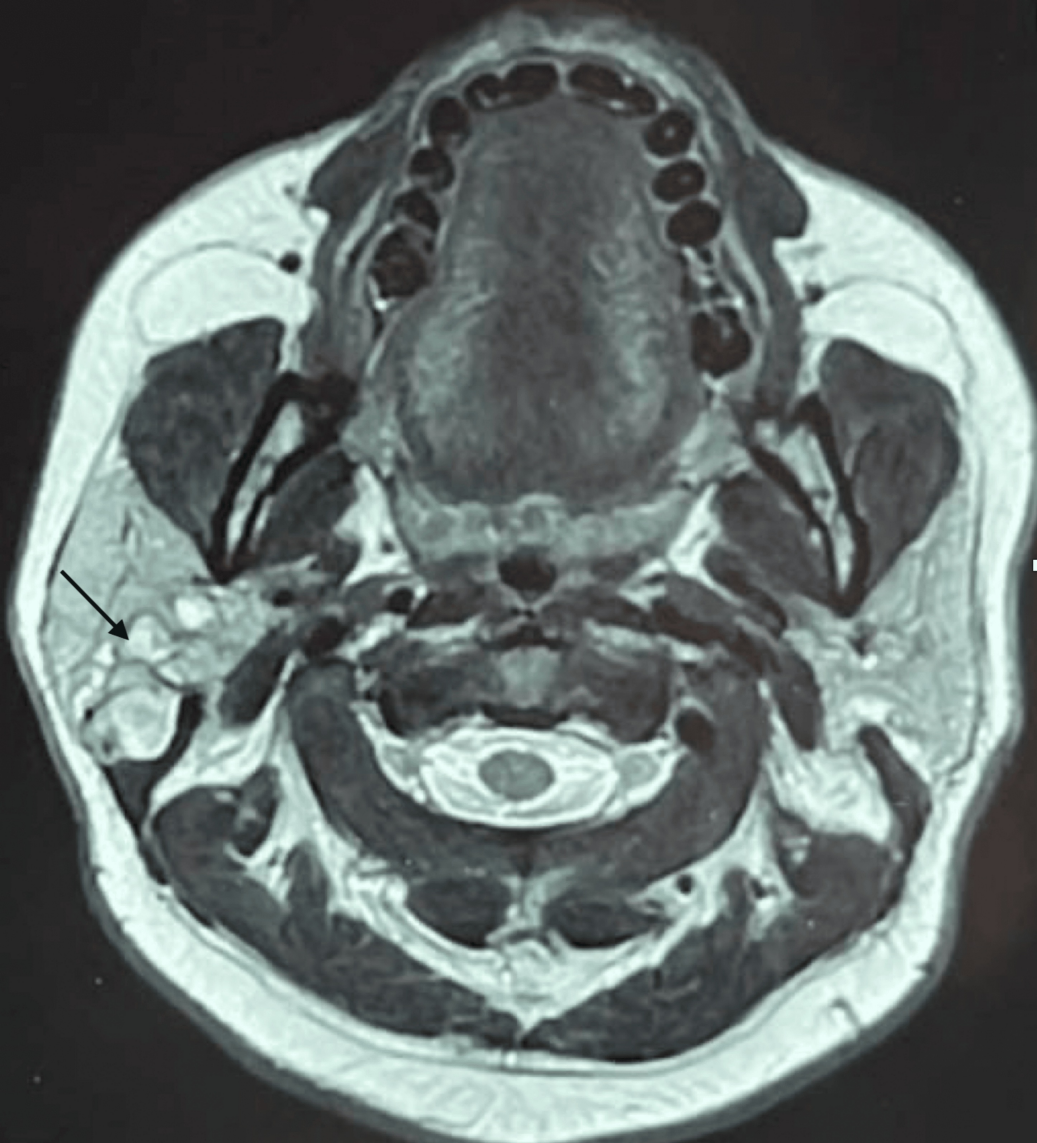
Axial MRI images showing a well-defined right parotid mass suggestive of a pleomorphic adenoma. The arrow indicates the parotid mass.

Clinical examination revealed a right lateral facial mass, 4 cm in greatest dimension, located in the preauricular area. The mass was painless, firm, non-pulsatile, of hard consistency, non-inflammatory, and fixed to deep planes. The Stensen’s duct orifice was patent, with no purulent or bloody discharge. The rest of the examination showed no abnormalities, particularly with normal cervical lymph node areas, and no signs of peripheral facial paralysis. MRI of the parotid glands (Figure 1) suggested a pleomorphic adenoma of the right parotid gland, measuring 42 × 30 × 25 mm, with a central hemorrhagic component.

**Figure 2:**
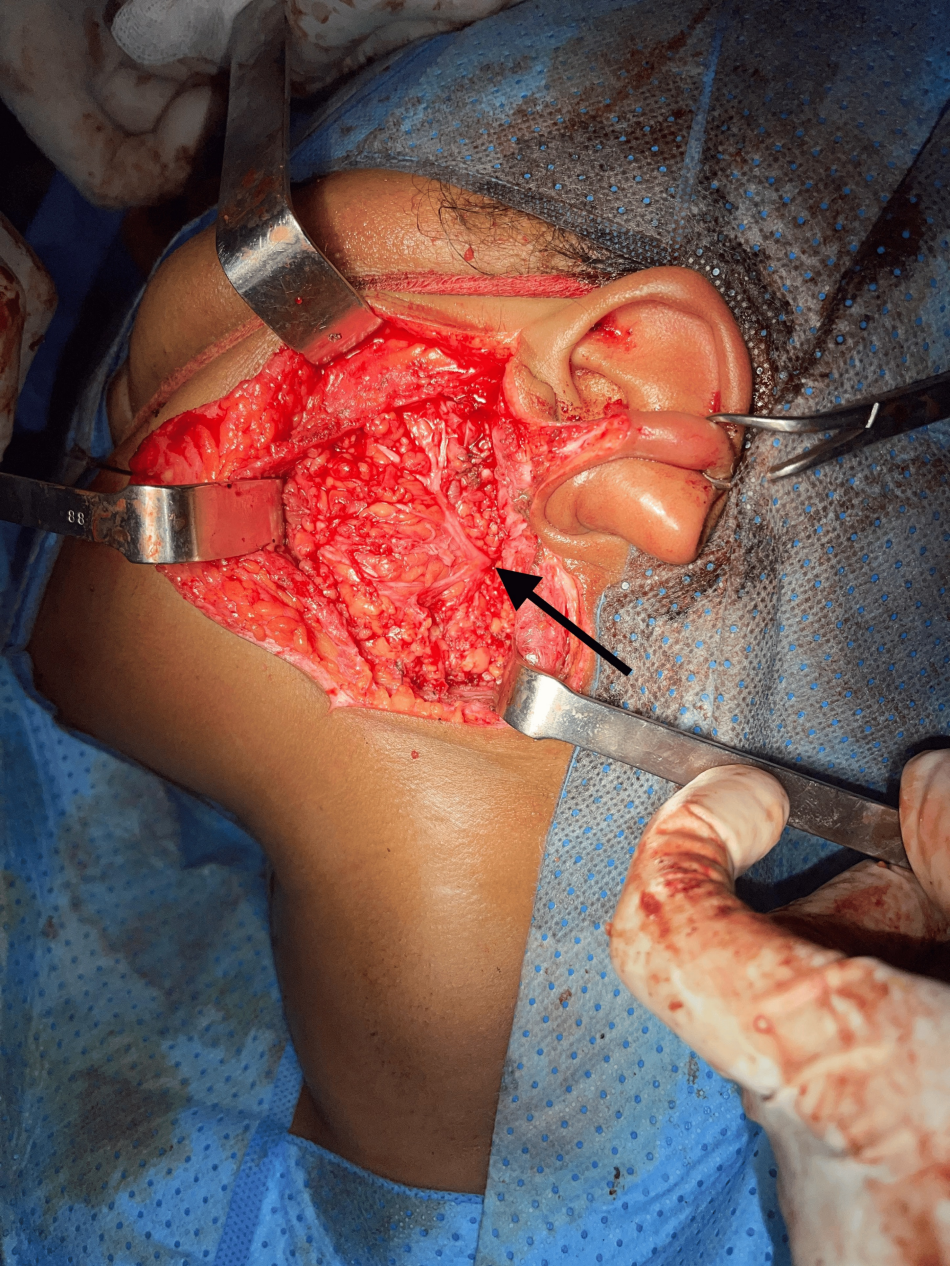
Intraoperative view of the right superficial parotidectomy. The arrow indicates the facial nerve and its main branches.

The patient underwent a right superficial parotidectomy (Figure 2) with an intraoperative frozen section, which revealed an indeterminate lesion with suspicion of malignancy.

**Figure 3:**
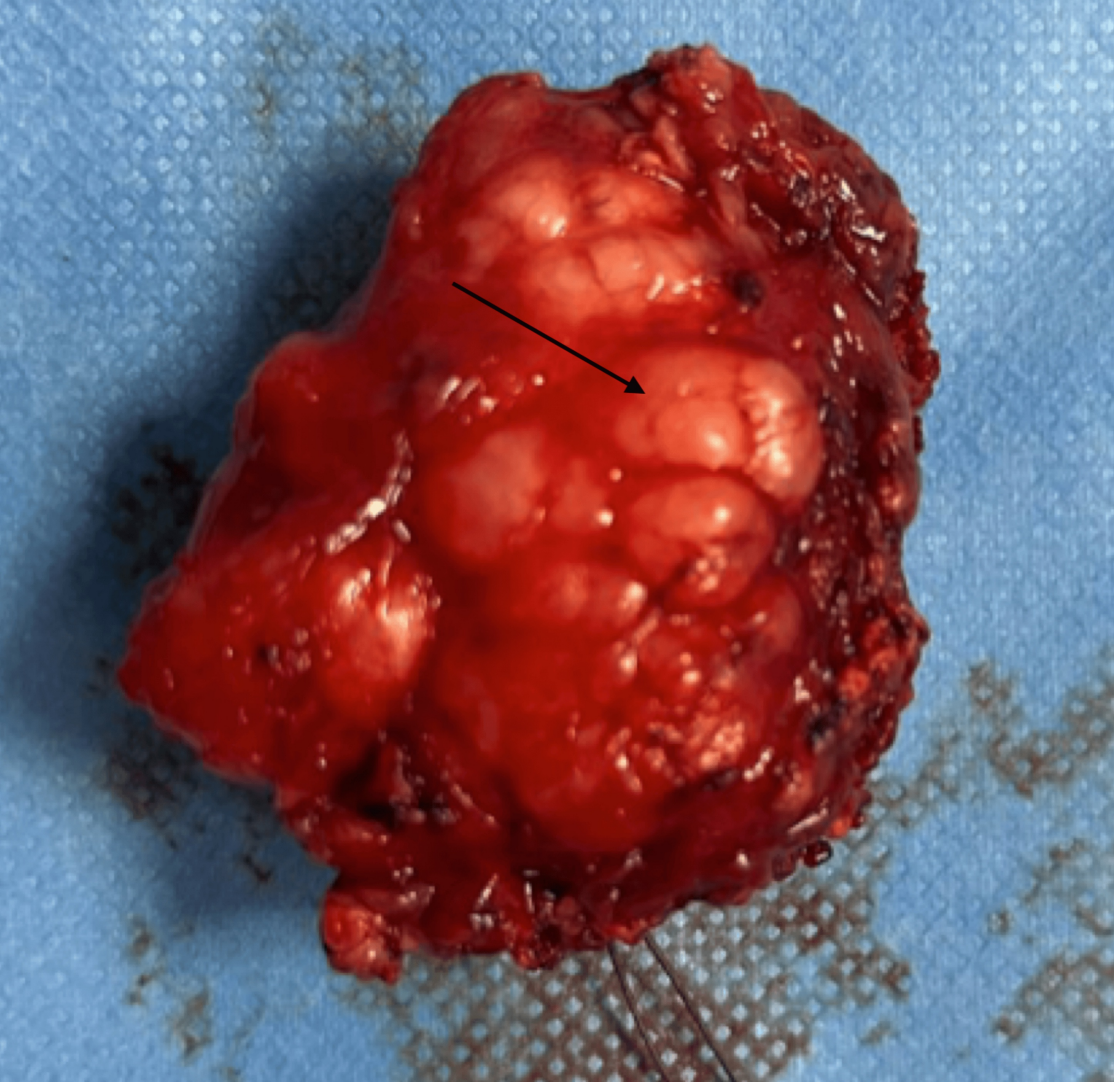
Gross specimen of the excised parotid gland after total parotidectomy. The arrow highlights the nodular lesion consistent with sclerosing polycystic adenosis.

A total right parotidectomy was subsequently performed (Figure 3).

Final histopathological examination revealed a well-circumscribed but non-encapsulated lesion composed of abnormal glandular structures arranged in a dense, sclerotic stroma with numerous variably sized cystic cavities, often filled with amorphous eosinophilic secretions, consistent with sclerosing polycystic adenosis of the right parotid gland. No signs of malignancy were observed.

The immediate and long-term postoperative follow-up showed no complications. The outcome was favorable, with good local healing, no wound infection, appropriate daily care, suture removal on day 10, and no peripheral facial paralysis or Frey’s syndrome.

## Discussion

Pleomorphic adenoma remains the most frequently encountered benign salivary gland tumor, predominantly affecting the parotid gland [4]. However, sclerosing polycystic adenosis (SPA) is a rare benign entity first described in 1996, exhibiting histopathological similarities with benign breast lesions such as sclerosing adenosis and complex mastopathy [1]. These similarities may result in diagnostic confusion, particularly with low-grade carcinomas, requiring careful histopathological and immunohistochemical evaluation for accurate diagnosis.

SPA generally affects young adults in the third decade of life, with no sex predilection [2,5]. Most tumors are localized in the parotid gland, although submandibular and accessory salivary glands may also be involved [1]. Lesions usually present as slow-growing, solitary, well-circumscribed, and sometimes partially encapsulated nodules ranging from 0.3 to 6 cm. Clinical symptoms are nonspecific and often consist of a painless mass; however, some patients may report pain, as observed in our case. Incidental findings of SPA during surgery for unrelated parotid conditions have also been reported [6].

The etiopathogenesis of SPA remains uncertain. Proposed contributing factors include recurrent parotitis, developmental ductal anomalies, certain systemic diseases, and immunodeficiency [2]. Histologically, SPA is characterized by collagenous sclerosis surrounding dilated ducts and cystic spaces filled with eosinophilic material, with associated lymphocytic infiltrates [3,5]. Immunohistochemistry is particularly valuable in differentiating SPA from malignancy; the presence of a myoepithelial cell layer, demonstrated by smooth muscle actin or other markets, supports a benign nature [1].

Although malignant transformation has not been documented, the rarity of SPA and the small number of reported cases mean this risk cannot be entirely excluded [1]. Differential diagnosis includes other benign tumors such as pleomorphic adenoma and Warthin’s tumor [7], and when located in the parotid gland, SPA may pose a risk to the facial nerve due to its proximity [8].

The treatment of choice is complete surgical excision, typically via conservative subtotal parotidectomy. This approach helps minimize recurrence, which can result from incomplete excision. Long-term follow-up is advised due to the potential for delayed recurrences and the theoretical risk of malignant transformation [1,2]. Postoperative facial nerve dysfunction is the most frequent complication, usually transient, but can be permanent in a small proportion of cases [8].

In the event of recurrence, repeat surgery or adjuvant radiotherapy may be considered [8].

## Conclusions

Sclerosing polycystic adenosis (SPA) of the parotid gland is a rare benign salivary gland tumor, with histological similarities to sclerosing adenosis of the breast.

Complete surgical excision is the treatment of choice to minimize the risk of recurrence or progression. Regular follow-up is recommended.
